# Intelligent Control of Groundwater in Slopes with Deep Reinforcement Learning †

**DOI:** 10.3390/s22218503

**Published:** 2022-11-04

**Authors:** Aynaz Biniyaz, Behnam Azmoon, Zhen Liu

**Affiliations:** Department of Civil, Environmental, and Geospatial Engineering, Michigan Technological University, Houghton, MI 49931, USA

**Keywords:** deep reinforcement learning, Deep Q-Network, landslide, intelligent control, seepage analysis, slope stability analysis

## Abstract

The occurrence of landslides has been increasing in recent years due to intense and prolonged rainfall events. Lowering the groundwater in natural and man-made slopes can help to mitigate the hazards. Subsurface drainage systems equipped with pumps have traditionally been regarded as a temporary remedy for lowering the groundwater in geosystems, whereas long-term usage of pumping-based techniques is uncommon due to the associated high operational costs in labor and energy. This study investigates the intelligent control of groundwater in slopes enabled by deep reinforcement learning (DRL), a subfield of machine learning for automated decision-making. The purpose is to develop an autonomous geosystem that can minimize the operating cost and enhance the system’s safety without introducing human errors and interventions. To prove the concept, a seepage analysis model was implemented using a partial differential equation solver, FEniCS, to simulate the geosystem (i.e., a slope equipped with a pump and subjected to rainfall events). A Deep Q-Network (i.e., a DRL learning agent) was trained to learn the optimal control policy for regulating the pump’s flow rate. The objective is to enable intermittent control of the pump’s flow rate (i.e., 0%, 25%, 50%, 75%, and 100% of the pumping capacity) to keep the groundwater close to the target level during rainfall events and consequently help to prevent slope failure. A comparison of the results with traditional proportional-integral-derivative-controlled and uncontrolled water tables showed that the geosystem integrated with DRL can dynamically adapt its response to diverse weather events by adjusting the pump’s flow rate and improve the adopted control policy by gaining more experience over time. In addition, it was observed that the DRL control helped to mitigate slope failure during rainfall events.

## 1. Introduction

Slope failures with the movement of massive soil, mud, or rock volumes displace thousands of people annually [1]. Although the global fatality rate from landslides is not well quantified, 2620 reported landslides between 2004 and 2010 caused 32,322 casualties worldwide [2]. Economic loss in such disasters is also considerable, as the annual cost of damages to infrastructure is estimated to be over USD 250 billion globally [3]. Extreme rainfall events are one of the main triggers for landslides [4]. In Central America and the Caribbean, for example, heavy rainfall was the cause of approximately 90% of the landslides recorded in the Enhanced Durham Fatal Landslides Database (EDFLD) [5]. Prolonged intense rainfall events reduce the stability of the slope by increasing the groundwater table and the degree of saturation above the groundwater table and consequently decreasing the unsaturated soil shear strength [6,7,8,9]. The frequency of heavy rainfall events has escalated in recent years as a result of climate change, which has increased the likelihood of landslide occurrence in general [10,11,12].

In order to control groundwater tables in real time and reduce the risk of landslides, subsurface drainage systems have been widely used in flood-prone locations [13,14,15]. Most drainage systems are designed to use the force of gravity for collecting water [15]. In the absence of gravity, subsurface drainage wells equipped with pumping systems are required to lower the groundwater [16,17]. However, inefficient manual operations of the pumping systems can significantly increase the labor and energy costs. In most cases, these high operational costs may only allow short-term usage of pumping systems [18]. This study aims to minimize the operational cost of such systems by boosting the autonomy of geosystems utilizing deep reinforcement learning (DRL).

DRL is a combination of deep learning and reinforcement learning (RL). Deep learning algorithms have been widely adopted in the field of geotechnical engineering for landslide detection [19,20], landslide susceptibility analysis [21,22], soil and rock classification [23,24], tunnel construction [25,26], and groundwater level prediction [27,28,29,30]. However, RL, which is a subfield of machine learning for automated sequential decision-making, has only been utilized in a few research studies in geotechnical engineering for tunneling applications [31,32,33]. Integration of the recent advances in deep neural networks with RL enabled DRL for revolutionary sequential decision-making in autonomous systems with high-dimensional state spaces [34,35,36]. Different studies showed the remarkable performance of DRL in games [34,36,37,38,39] and real-world applications such as robotics [40], autonomous driving [41], the control of stormwater systems [42], and carbon storage reservoir management [43]. Therefore, this study investigates the concept of using DRL for the intelligent control of groundwater. This concept can help to generate an intelligent geosystem that can adjust the pump’s flow rate and control the groundwater table in response to dynamic rainfall intensity. To the best of our knowledge, we are the first to implement DRL for controlling the groundwater in slopes.

This paper is an extended version of our preliminary study on developing a DRL framework for the intelligent control of groundwater in a typical geosystem (i.e., a slope equipped with a pump and subjected to rainfall events) [44]. The main contributions of this paper are (1) modifying the DRL framework proposed in our previous study [44], such as the reward function and training hyperparameters; (2) evaluating the DRL control of the water level against the traditional proportional-integral-derivative (PID)-controlled and uncontrolled water levels; (3) assessing the performance of DRL control in preventing slope failures; (4) investigating the effectiveness of transferring the DRL agent’s knowledge from a pre-trained model to a new training task with a different rainfall event; (5) exploring the influence of the number of observations from the environment, and (6) investigating the impact of binary control versus intermittent control on the groundwater management.

The remainder of the paper is structured as follows. Section 2 first reviews the fundamentals of RL in the geosystem and then introduces the environment, agent, and reward function. Section 3 explains how the performance of the DRL for groundwater control was evaluated for various rainfall events. Section 4 provides settings and results for training the deep neural network in the DRL. Section 5 and Section 6 present more in-depth discussions and conclusions, respectively.

## 2. Deep Reinforcement Learning for Geosystems

### 2.1. Basics of Reinforcement Learning for Geosystems

**Figure 1:**
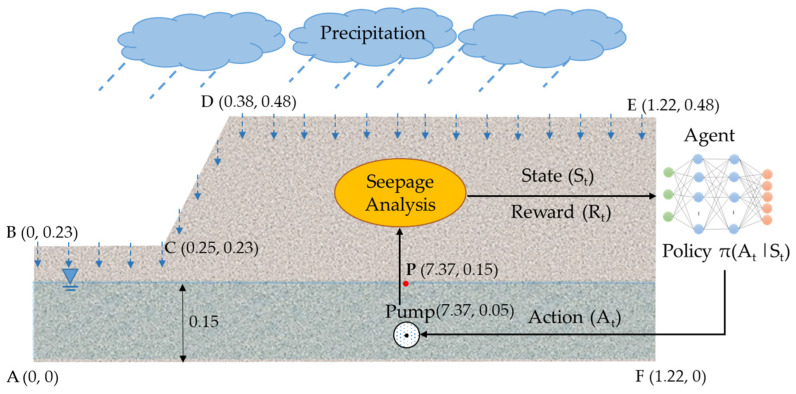
Agent–environment interaction in the geosystem; the virtual learning environment consists of a lab-scale slope equipped with a pump and subjected to precipitation (unit: m).

In the control enabled by RL, the (learning) agent interacts with an unknown environment to explore the optimum control policy that maximizes the cumulative reward [45,46]. The agent takes an action depending on the state of the environment, and the environment responds by returning the next state and a reward to evaluate the agent’s performance. The agent learns from the “trial and error” process, starting with random actions, and then, over time, it learns which action can return long-term rewards [47]. The Markov Decision Process (MDP) allows us to represent the above agent–environment interaction in a mathematical framework [43]. Figure 1 shows the agent–environment interaction and the geometry of the lab-scale geosystem.

The basic elements for RL in the geosystem are as follows.

- Environment: In this study, the RL environment is the lab-scale geosystem simulated with a numerical model for seepage. The seepage model informs the agent on the geosystem’s condition and specifies what state it can be in after performing an action. In future real-world applications, this environment can be the geosystem and its surrounding environment in the field. Simulation of the geosystem using a seepage model is thoroughly discussed in Section 2.2.
- Agent: The RL agent works as a pump operator in the RL framework. More specifically, it embodies the neural network algorithm that controls the water table by observing the current state of the geosystem and taking actions to regulate the pump’s flow rate. In this study, a Deep Q-Network (DQN) was adopted as the learning agent, which is covered in depth in Section 2.3.
- State ($S_{t}$): The state describes the current condition of the environment (i.e., the geosystem). In this study, the RL agent receives three observations from the environment before taking an action. The observations are (1) the water head at point “P” in Figure 1 representing the distance from the target level, (2) the rain intensity at the current time step, and (3) the rain intensity at the next time step. A transient seepage analysis was performed at each time step to determine the water head at point “P”.
- Action ($A_{t}$): An action is an operation taken by the agent in the current state. For this geosystem, an action was considered to control the pump’s flow rate for each time step. The action space contains all the possible actions that the agent can take. To enable intermittent control of the geosystem, five discrete actions were defined, $A_{t}=[0,1,2,3,4]$, representing 0%, 25%, 50%, 75%, and 100% of the pumping capacity, respectively.
- Reward ($R_{t}$): The reward is the evaluation score or feedback assigned to the agent for its action. At any given time t, the agent observes the state of the geosystem, and then, based on this, takes an action to regulate the pump’s flow rate for controlling the water level. Subsequently, the agent receives a reward to assess the action choice. The reward function is defined to designate the desired and undesired actions in the current state. The agent will receive a positive reward if the action can keep the groundwater close to the target level. If the groundwater moves away (up or down) from the target level, the agent will receive a negative reward related to the distance of the water table from the target level.

### 2.2. Environment Simulation: Seepage Model

A seepage model was employed to simulate the geosystem (i.e., a slope equipped with a pump and subjected to rainfall events). To update the agent in the state $S_{t}$, a transient seepage analysis was conducted with the model to determine the water head at point “P” for the given rain intensity at time t. The seepage analysis was carried out using DOLFIN [48], the Python interface of FEniCS [49]. FEniCS is an open-source library for solving partial differential equations (PDEs). In our previous study [50], a similar seepage model was seamlessly coupled with a slope stability analysis to investigate the influence of the water level fluctuation in the reservoir on the stability of silty and sandy slopes. The computational framework of the seepage model was validated using another finite element PDE solver, FlexPDE [50]. The present seepage model, however, differs in the boundary conditions, soil properties, and slope geometry. The governing equation for the saturated–unsaturated transient seepage analysis in this study is given in Equation (1).

(1) $$S\frac{\partial (h+z)}{\partial t}=K\times \nabla \left(\nabla \left(h+z\right)\right)+q_{s},$$

where h [m] is the pressure head, z [m] is the elevation head, and $q_{s}$ [m/s] is the sink term representing the pump’s outflux.

The definitions of the terms S and K depend on the soil’s degree of saturation. In a saturated flow, S and K were replaced with $S_{s}$ (specific storage of saturated flow) and $K_{s}$ (saturated hydraulic conductivity), respectively. $S_{s}$ and $K_{s}$ were determined based on the type of soil and were assumed to be constant during the transient seepage analysis. In an unsaturated flow, S and K were substituted by $S_{c}$ (specific moisture content) and $K_{s}K_{r}$, where $K_{r}$ is the relative hydraulic conductivity. The specific moisture content for the unsaturated flow is the derivative of volumetric water content (θ) with respect to the water head (h),

(2) $$S_{c}=\left|\frac{\partial \theta }{\partial h}\right|=n\frac{\partial S_{e}}{\partial h},$$

where n [−] is the soil porosity and $S_{e}$ is the effective saturation degree. $S_{e}$ was derived from the van Genuchten equation [8,51],

(3) $$S_{e}={\left[1+{\left(\frac{\psi }{P_{0}}\right)}^{\frac{1}{1-a}}\right]}^{-a},$$

where a and $P_{0}$ [Pa] are fitting parameters that can be obtained from the soil–water characteristic curve (SWCC). ψ [Pa] is the matric suction and is calculated as follows:

(4) $$\psi =\gamma_{w}\left|h\right|,$$

where $\gamma_{w}$ [N/m^3^] is the unit weight of water.

The relative hydraulic conductivity quantifies how the hydraulic conductivity changes with the degree of saturation. The widely adopted van Genuchten equation was used for $K_{r}$ [51]:

(5) $$K_{r}=S_{e}^{0.5}{\left(1-{\left(1-S_{e}^{1/a}\right)}^{a}\right)}^{2},$$

Table 1 presents the input parameters adopted for the seepage model. These parameters were obtained based on a combination of lab tests and published ranges of values for similar soils from the literature.

The geometry of the geosystem used in the analyses is shown in Figure 1. In fact, the physical counterpart of this lab-scale geosystem will serve as a real-world environment in future studies for testing the proposed methodology in this study. The lab-scale geosystem was located in an acrylic tank to control the influx and outflux in the system. Accordingly, the no-flux boundary condition was assigned to the bottom (“AF”), left (“AB”), and right (“FE”) sides of the slope.

(6) $$-\nabla \left(h+z\right)\cdot \vec{n}=0\;\mathrm{on}\;\Gamma_{\left(\mathrm{AF},\;\mathrm{AB},\;\mathrm{FE}\right)}\;\mathrm{for}\;t>0,$$

**Figure 2:**
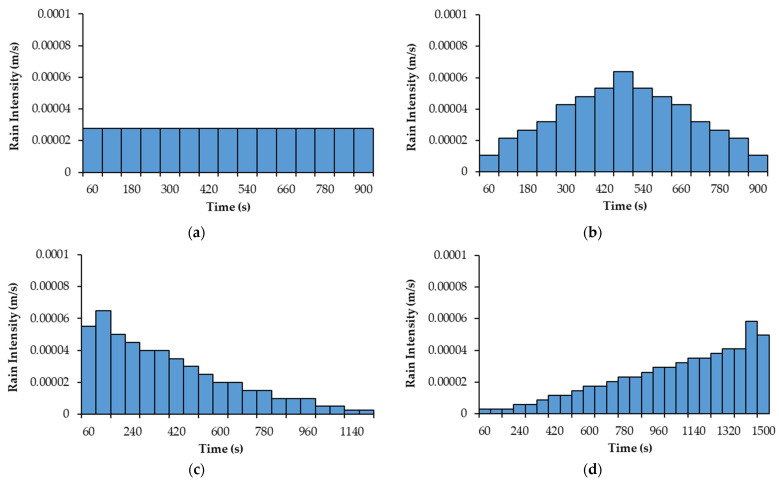
Rain intensity distributions of rainfall events with various patterns, durations, and total rainfall depths: (**a**) 15 min-constant; (**b**) 15 min-normal; (**c**) 20 min-descending; (**d**) 25 min-ascending.

The only influx into the geosystem is the rain infiltrating across the slope’s top surfaces (“BC”, “CD”, and “DE”):

(7) $$-\nabla \left(h+z\right)\cdot \vec{n}=I_{r}\;\mathrm{on}\;\Gamma_{\left(\mathrm{BC},\;\mathrm{CD},\;\mathrm{DE}\right)}\;\mathrm{for}\;t>0,$$

where $I_{r}$ [m/s] is the rain intensity [m/s]. Four different rainfall events, as shown in Figure 2, were considered to train the agent. The rainfall events were designed based on three parameters: (1) rainfall duration, (2) total rainfall depth, and (3) rain intensity distribution pattern. For rain intensity distribution patterns, German guidelines, Deutschen Verbandes für Wasserwirtschaft und Kulturbau (DVWK), recommend four possible intensity distribution patterns for rainfall events, as displayed in Figure 2 [52]. Accordingly, rainfall events with various durations (15, 20, 25 min), total rainfall depths (25 mm, 30 mm, 32 mm, and 35 mm), and patterns (constant, normal, descending, and ascending) were used in the seepage model. Figure 2a is a 15-min event with a constant rain intensity and a total rainfall depth of 25 mm. Figure 2b is a 15-min event with a maximum intensity in the middle of the event and a total rainfall depth of 32 mm. Figure 2c is a 20-min event with a maximum intensity at the beginning of the event and a total rainfall depth of 30 mm. Figure 2d is a 25-min event with a maximum intensity at the end of the event and a total rainfall depth of 35 mm. For simplicity, these events will be referred to as “15 min-constant”, “15 min-normal”, “20 min-descending”, and “25 min-ascending”, respectively. It is noted that the water ponding was not considered in the seepage analysis since the rain intensity in all four events was smaller than the saturated hydraulic conductivity (i.e., soil infiltration capacity).

The pump was modeled as a sinkhole in the analyses. Thus, an outflux boundary condition was set to the pump’s boundary. The pump’s outflux is the discharge per unit area per unit time [m^3^/m^2^s or m/s].

(8) $$-\nabla \left(h+z\right)\cdot \vec{n}=\chi \frac{Q_{p}}{2\pi r}\;\mathrm{on}\;\Gamma_{\left(\mathrm{Pump}\right)}\;\mathrm{for}\;t>0,$$

where $Q_{p}$ [m^3^/s] is the maximum capacity of the pump and r [m] is the radius of the sinkhole for the pump. χ [–] takes a value between 0 and 1 depending on the action taken by the agent to regulate the pump’s flow rate. Five discontinuous actions were defined for the agent to set the pump’s capacity to 0%, 25%, 50%, 75%, and 100% of the maximum capacity. Table 2 presents the values of $Q_{p}$, r, and χ adopted for the five actions.

**Figure 3:**
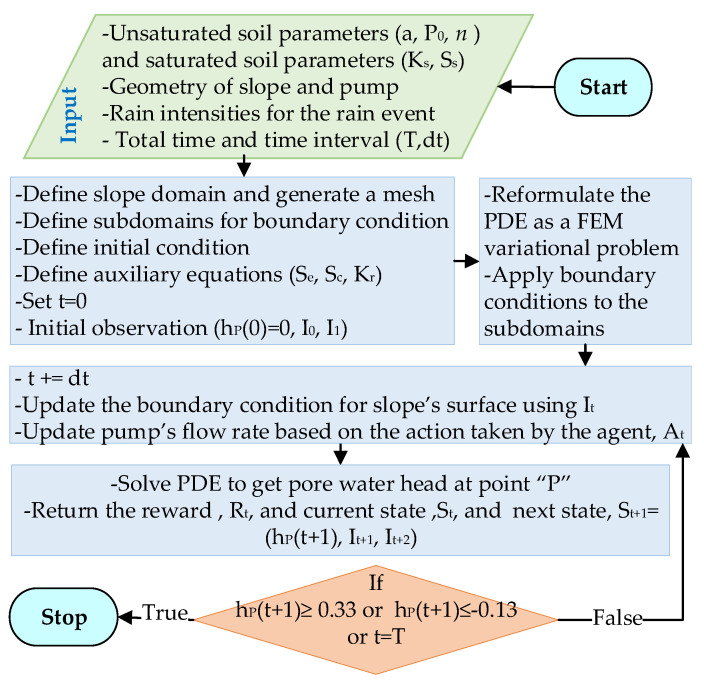
Flowchart of the developed Python code for the seepage model (i.e., virtual environment).

Figure 3 shows the flowchart of the developed Python code for the seepage model. The input parameters for this model are unsaturated soil characteristics ($a,P_{0},n$), saturated soil parameters ($S_{s},K_{s}$), the geometry of the slope and pump, the location of point “P”, rain intensities for the rainfall event ($I_{r}$), and time variables (T,dt). T is the duration of the rainfall event and dt is the time step for solving the PDE (i.e., the governing equation). The time step was considered 1 min. In the next step, the computational domain of the slope was defined and a mesh with 3-node Lagrangian elements was generated. Next, the subdomains, the initial water level, the initial observations (i.e., initial water head and rain intensity at *t* = 0, 1 min), and the auxiliary equations ($S_{e},S_{c},K_{r}$) were defined. In order to solve the PDE, the equation was reformulated as a finite element variational problem. Boundary conditions were then applied to the subdomains, as demonstrated in Equations (6)–(8). For each time step, the boundary conditions for the slope’s surface (“BC”, “CD”, and “DE” in Figure 1) and the pump were updated based on the rain intensity and the action taken by the agent. Subsequently, the PDE was solved to obtain the water head at point “P”. The result was used to calculate the reward and update the agent on the next state. The details about the reward function and conditions for terminating the seepage analysis will be explained in Section 2.4.

### 2.3. Agent: Deep Q-Network

The Deep Q-Network (DQN) is a widely accepted algorithm for sequential decision-making in systems with high-dimensional states. DQN was introduced in 2015 by combining the Q-learning algorithm and deep neural networks (DNNs), which showed human-level performance in playing Atari games [34]. Recent DQN studies also demonstrated great success in controlling complex systems in a variety of disciplines. Successful examples of DQN applications include real-time control of stormwater systems [42], carbon storage reservoir management [43], stock market forecasting [53], managing health care system [54], control of agricultural irrigation [55], and crop yield prediction for sustainable agrarian applications [56]. DQN is the ideal option for automated decision-making in the geosystem because it has shown excellent performance in systems with high-dimensional states.

DQN is a value-based algorithm. This means that, for the given state, DQN assigns a state–action value (i.e., $Q^{*}\left(S_{t},A_{t}\right)$), to each possible action as follows [34]:

(9) $$Q^{*}\left(S_{t},A_{t}\right)=Q\left(S_{t},A_{t}\right)+\alpha \left[R_{t}+\gamma \max Q\left(S_{t+1},A_{t+1}\right)-Q\left(S_{t},A_{t}\right)\right],$$

where $R_{t}$ is the immediate reward in response to the action $A_{t}$ taken in the state $S_{t}$, $\max Q\left(S_{t+1},A_{t+1}\right)$ is the maximum *Q*-value in the next state $S_{t+1}$ after taking the (optimum) action $A_{t+1}$, α is the learning rate of the agent, and γ is the discount factor. The learning rate is a hyperparameter between 0 and 1 (0<α≤1), which determines the step size of the update for *Q*-values. The condition α=0 overlooks the knowledge from new actions and does not update the *Q*-value, while α=1 considers the most recent information and ignores the acquired knowledge from the past. The discount factor takes a fixed value between 0 and 1 (0≤γ≤1) to adjust the contribution of long-term rewards from future states and actions. In fact, γ=0 merely considers the immediate reward for the action $A_{t}$ and ignores the future outcomes of the chosen actions, while γ=1 evaluates actions equally based on their immediate reward and potential future rewards [46].

**Figure 4:**
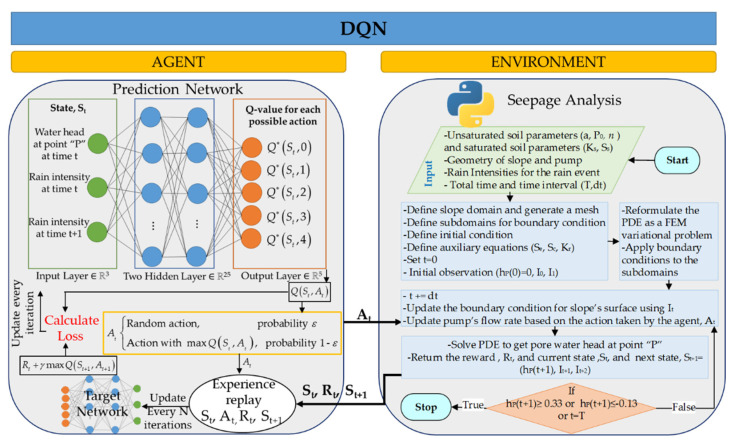
Architecture of the proposed DQN model.

The *Q*-values are initialized with random values because the agent does not have any knowledge about the environment. When the agent starts to take action, the *Q*-values are continuously updated using Equation (9) until converging to an optimal policy. As the state and action space size increase, a neural network helps to approximate the *Q*-values, leading to DQN. Figure 4 demonstrates the architecture of the DQN model for the current study and the interactions between the environment (i.e., the seepage model) and the agent (i.e., the neural network).

In the DQN model shown in Figure 4, the agent takes an action based on the ε-greedy policy. The ε-greedy policy helps the agent to strike a balance between exploitation and exploration [57,58]. Exploitation is a strategy in which the agent greedily chooses the most effective previously discovered action, whereas exploration allows the agent to explore its environment by taking random actions that may occasionally return even higher rewards [42]. Based on this policy, the agent randomly chooses an action with the probability of ε or takes a known action associated with the maximum *Q*-value with the probability of 1−ε (see Equation (10)). At the beginning of the training, it is common to set ε to a high value (e.g., 1) to enable the agent to explore the environment for rewarding actions. This parameter is gradually reduced to a lower value (e.g., 0.01) to transition to an exploitation strategy as the agent converges to an optimal control policy [42,43].

(10) $$Loss={\left[\underset{\mathrm{Target}\;\mathrm{Q-value}}{\underset{︸}{\left(R_{t}+\gamma \max Q\left(S_{t+1},A_{t+1}\right)\right)}}-\underset{\mathrm{Predicted}\;\mathrm{Q-value}}{\underset{︸}{Q\left(S_{t},A_{t}\right)}}\right]}^{2},$$

As shown in Figure 4 for the DQN model, two separate neural networks with the same architecture were trained simultaneously to stabilize the learning process [34]. The first network is the prediction network used to approximate the $Q\left(S_{t},A_{t}\right)$. The second network is the target network used to calculate the target *Q*-values (i.e., future rewards), $\left(R_{t}+\gamma \max Q\left(S_{t+1},A_{t+1}\right)\right)$. The input layer of each neural network contains three neurons to receive observations from the environment. Two fully connected hidden layers were defined, with 25 neurons for each layer. Then, the output layer with five neurons was specified for *Q*-values of five possible actions. Mnih et al. also introduced the experience replay (or replay buffer) to improve the learning stability [34]. The experience replay stores the agent’s most recent experience as a tuple of ($S_{t}$,$A_{t}$,$R_{t}$,$S_{t+1}$). During the training, the agent samples a batch of data from the experience replay and calculates the loss of the neural network, and then updates the prediction network weights. The loss function is the squared difference between the target *Q*-value and the predicted *Q*-value:

(11) $$Loss={\left[\underset{\mathrm{Target}\;\mathrm{Q-value}}{\underset{︸}{\left(R_{t}+\gamma \max Q\left(S_{t+1},A_{t+1}\right)\right)}}-\underset{\mathrm{Predicted}\;\mathrm{Q-value}}{\underset{︸}{Q\left(S_{t},A_{t}\right)}}\right]}^{2},$$

It is noted that both prediction and target networks were initialized with the same weights. The weights for the prediction network were updated every iteration, whereas the weights for the target network were updated every N iterations (e.g., every 50 iterations) to stabilize the training. The target network simply duplicates the weights of the prediction network every N iterations. For this study, after trying various values (15, 45, 60, and 75) of target network update frequency (or N), the value of 60 was selected.

### 2.4. Reward Function

The performance of the DRL agent is highly dependent on the received rewards during the training [38]. A thorough and explicit reward function can assist the agent in rapidly discovering the optimal policy and achieving the goal of the system. However, outlining such a reward function is not a simple task. In this study, the reward function was defined in such a way as to incentivize the agent to adopt a control policy that keeps the water level close to the target level. The reward function for the geosystem (Equation (12)) was constructed using the absolute value of the water head at point “P” at the next time step (i.e., $\left|h_{P}(t+1)\right|$), and the difference between the water head values at point “P” at the current and next time steps (i.e., $h_{P}(t+1)-h_{P}(t)$). The positive and negative values of the water head at point “P” represent the water levels above and below point “P”, respectively. The absolute value of the water head represents the distance between the current and the target water level.

(12) $$R_{t}=\left\{ \begin{array}{ll} 100-90\left[\left|h_{P}(t+1)\right|/0.01\right], & \left|h_{P}(t+1)\right|\leq 0.01\;\\ 1000\left(\left|h_{P}(t+1)-h_{P}(t)\right|\right), & \left|h_{P}(t+1)\right|\leq \left|h_{P}(t)\right|\\ 1000\left(h_{P}(t+1)-h_{P}(t)\right), & h_{P}(t+1)>h_{P}(t)\&A_{t}=4\\ -10\left[\log_{10}\left(\left|h_{P}(t+1)\right|*100\right)\right], & \;\mathrm{else} \end{array}\right.,$$

The cumulative reward for an episode can help to evaluate the selection of actions for a rainfall event. An episode is a period in which the agent takes action in response to a rainfall event. An episode may last the same amount of time as a rainfall event. In addition to the episode duration, in this study, an episode was terminated when there was an overflow or complete discharge in the slope. The overflow would happen when the water height above point “P” exceeds 0.33 m, and the complete discharge would occur when the water level is more than 0.13 m below the target level. Terminating an episode due to an overflow and a complete discharge leads to a lower cumulative reward, so the agent would attempt to avoid these situations by refining the adopted policy. Such settings can benefit both the geosystem and the pump by enhancing the safety and efficiency of the system.

The geosystem’s goal was to keep the water level as close as possible to the target level. It is noted that the geosystem was initially set to the target level. By starting the precipitation, the water level gradually increased and the agent began taking action. The agent received a positive score for reducing the distance to the target level (i.e., $\left|h_{P}(t+1)\right|\leq \left|h_{P}(t)\right|$). The reward function assigned a higher reward to actions that led to lower absolute values of water head at point “P” (i.e., $\left|h_{P}(t+1)\right|\leq 0.01$). The agent also earned a positive score when the pump utilized its full capacity to remove water from the slope; however, it could not reduce the distance from the target level. The maximum possible reward value that the agent could receive for each action was 100, which happened when the water head at point “P” was exactly zero. By contrast, the agent received a negative score when the water level moved away from the target level. The maximum negative score assigned to the agent was approximately −15, which happened when the water level approached the overflow level.

## 3. Performance Evaluation

The performance of the proposed DRL in controlling the groundwater was evaluated by comparing the variation in the water level achieved with the DRL control to the water levels obtained (1) with no control and (2) with a traditional control method called proportional-integral-derivative (PID). The uncontrolled water level represents the condition when no human intervention, e.g., pumping in this study, is applied. The uncontrolled water level in the slope during rainfall events was obtained by performing a transient seepage analysis with the no-flux boundary for the pump. PID is one of the most widely used control methods, which is introduced in Section 3.1.

In order to assess the effectiveness of each water control method in preventing slope failures, coupled transient seepage and slope stability analyses were seamlessly conducted to obtain the slope’s factor of safety (FS) during various rainfall events. The methodology for calculating the FS is reviewed in Section 3.2. The evaluation metric of root mean square error (RMSE) also helped to compare the performance of different methods in controlling the groundwater.

(13) $$\mathrm{RMSE}={\left[\frac{1}{n}\sum_{i=1}^{n}{\left(y_{i}-{\hat{y}}_{i}\right)}^{2}\right]}^{\frac{1}{2}},$$

where n is the total number of time steps. For each time step, $y_{i}$ is the target value, and ${\hat{y}}_{i}$ is the water head at point “P” using different control methods. The RMSE takes a value within [0,+∞] and has the same unit as the variable of interest (i.e., water level). RMSE values close to 0 indicate the good performance of the method in controlling the groundwater [59].

### 3.1. PID Controlled Groundwater

**Figure 5:**
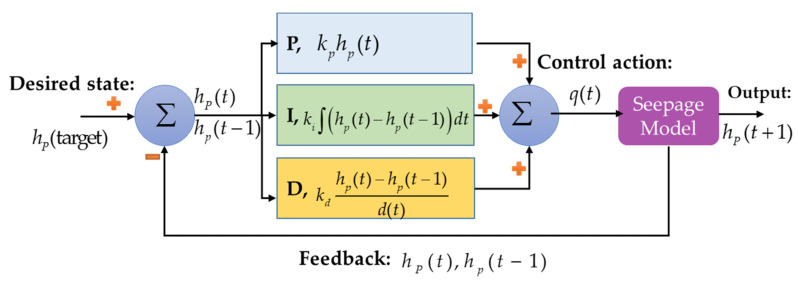
PID loop control for the geosystem.

PID control is one of the most common control algorithms due to its simple implementation and clear functionality [60,61]. This controller mainly calculates the error (i.e., the difference between the desired output and the actual output) and employs the proportion, integration, and derivation components of the error in the control function. The output of the PID control is formulated as follows [61]:

(14) $$u(t)=k_{p}e(t)+k_{i}\int e(t)dt+k_{d}\frac{de(t)}{dt}，$$

where is the PID-controlled variable, $k_{p}$ is the proportional gain, e(t) is the error value, $k_{i}$ is the integral gain, $k_{d}$ is the derivative gain, dt is the change in time, and de is the change in the error value. Figure 5 shows the PID control configuration for the geosystem in this study. In this configuration, q(t) is the control variable, which is the pump’s outflux. q(t) is a continuous value, so the type of control using PID is continuous. The error value is the difference between the current water head and the target water head at point “P”. Since the target water head at point “P” is zero, the $h_{P}(t)$ is the error value in this geosystem.

Equation (14) was updated based on the controlled variable and error value in the geosystem:

(15) $$q(t)=k_{p}h_{P}(t)+k_{i}\sum \left(h_{P}(t)-h_{P}(t-1)\right)dt+k_{d}\left(h_{P}(t)-h_{P}(t-1)\right)/dt$$

where dt is the time step for the seepage analysis (dt=60s), and $h_{P}(t)-h_{P}(t-1)$ represents the change in the error value (i.e., the difference between the water head values at point “P” at the current and previous time step). The PID parameters as listed in Table 3 were manually tuned. These parameters were tuned via a trial-and-error process that does not require any mathematical model [62]. In this process, the parameters $k_{i}$ and $k_{d}$ were initially set to zero. $k_{p}$ was then gradually increased until the output, started to oscillate. After fixing the $k_{p}$ value, $k_{i}$ was gradually increased. It was noticed that higher values of $k_{i}$ caused instability. Subsequently, $k_{d}$ was increased until an excessive response happened. These listed values of PID control parameters yielded the best results for the geosystem control problem investigated in this study.

### 3.2. Factor of Safety

Assessment of the slope’s FS during precipitation requires coupling the transient seepage analysis and the slope stability analysis seamlessly. The Python code developed in our previous study [50] was utilized for the slope stability analysis, which was then coupled with the seepage model of the geosystem. The FS was calculated using the Bishop Simplified method [63], a limit equilibrium method, which was modified with Vanapalli et al.’s [64] model to consider the unsaturated shear strength above the water table [50].

(16) $$FS=\sum_{j=1}^{N_{x}}\frac{1}{m_{a}m_{b}}\left[c^{\prime }+\left(m_{c}-U_{a_{j}}\right)\tan\phi^{\prime }+{\left(U_{a}-U_{w}\right)}_{j}\tan\phi^{\prime }S_{e_{j}}\right],$$

where $c^{\prime }$ [$\mathrm{kN}/m^{2}$] is the effective cohesion, $\phi^{\prime }$ [${}^{{}^{\circ}}$] is the effective internal friction angle, $S_{e_{j}}$ is the effective saturation at the base of the vertical slice *j*, and [$\mathrm{kN}/m^{2}$] is the matric suction at the base of the vertical slice *j*, in which $U_{a}$ is the pore air pressure and $U_{w}$ is the pore water pressure. $m_{a}$, $m_{b}$, and $m_{c}$ are defined as follows.

(17) $$m_{a}=\cos\alpha_{j}+\frac{1}{FS}\sin\alpha_{j}\tan\phi^{\prime },$$

(18) $$m_{b}=\frac{W_{j}\sin\alpha_{j}}{B_{x}}-\frac{{\left(F_{w}l_{p}\right)}_{j}}{RB_{x}},$$

(19) $$m_{c}=\frac{\left(W_{j}+F_{w_{j}}\cos\beta \right)}{B_{x}},$$

where $\alpha_{j}$ is the angle between the tangent to the base of the vertical slice *j* and the horizontal direction, $W_{j}$ is the total weight of the vertical slice *j*, $F_{w_{j}}$ is the hydrostatic force of the vertical slice *j* when the vertical slice is submerged, $l_{p}$ is the moment arm of $F_{w}$ about the center of the slip surface, R is the radius of circular slip surfaces, β is the angle between the slope and the horizontal direction, and $B_{x}$ is the width of the vertical slice j.

In the slope stability analysis, the outflux boundary for the embedded pump was changed depending on the type of control (i.e., DRL control, PID control, or no control). Model parameters for the slope stability analysis are presented in Table 4.

## 4. Network Training and Results

The proposed model was trained with multiple hyperparameter settings to find the optimal architecture of the DQN model for groundwater control. This process is called hyperparameter tuning. The model hyperparameters are parameters that influence the training process, such as the number of hidden layers for the deep neural network, learning rate, epsilon, and gamma. After completing the hyperparameter tuning, Table 5 shows the hyperparameter values that were optimized. The deep neural network was composed of four layers: one input layer with 3 neurons, two hidden layers with 25 neurons for each layer, and one output layer with 5 neurons. The input layer received three observations from the environment and the output layer generated *Q*-values for the five defined actions. Both the hidden layers and the output layer were fully connected layers (i.e., dense layers). A ReLU activation function [65] and linear activation function (also called no activation) were used for the hidden layers and the output layer, respectively. The adaptive Adam optimizer with a learning rate of 0.001 was adopted for training.

In order to assess the learning ability of DRL in controlling the groundwater during various rainfall events, the DQN model was trained four times in parallel with four different rainfall events, displayed in Figure 2. It is noted that the training for each rainfall event was independent. In other words, the model weights from training with a rainfall event were not transferred to another training process with a different rainfall event. The number of episodes for the training for each rainfall event was 10,000. An episode is a series of states, actions, and rewards that terminates when the rainfall period ends or when an overflow or a complete discharge occurs in the geosystem. During 10,000 episodes of training for each rainfall event, the agent interacts with the environment in an attempt to discover the policy that provides the highest cumulative rewards for each episode. Over the training period, the agent updates the model weights and improves the adopted groundwater control policy. After the training for all four rainfall events was complete, the DRL’s performance in controlling the groundwater table and regulating the pump’s flow rate during various rainfall events was evaluated.

**Figure 6:**
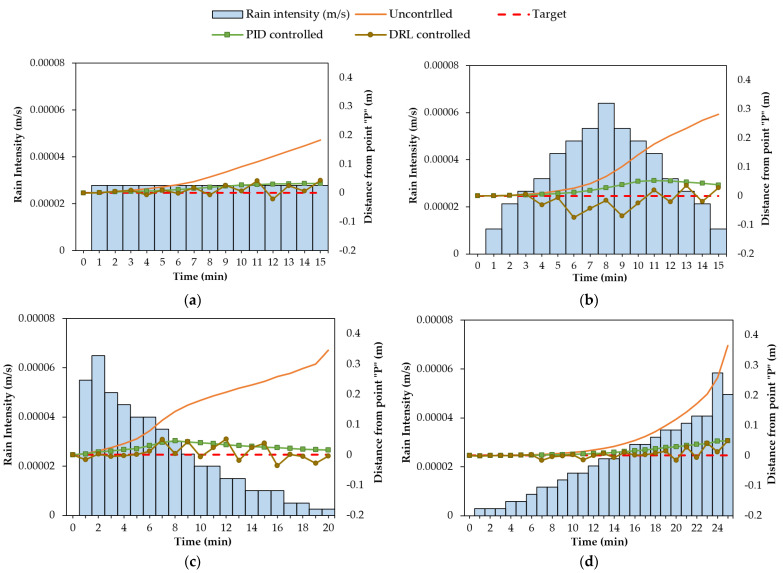
Distance of the water level from point “P” obtained with different control methods during rainfall events: (**a**) 15 min-constant; (**b**) 15 min-normal; (**c**) 20 min-descending; (**d**) 25 min-ascending.

Figure 6 shows the results for the water head variations at point “P” during four different rainfall events in the DRL-controlled, PID-controlled, and uncontrolled water levels. DRL’s performance in controlling the water level during each rainfall event was assessed by comparing it to the PID-controlled and uncontrolled water levels. During all rainfall events, the PID and DRL were able to regulate the pump’s flow rate and avoid overflow and complete discharge in the geosystem. RMSE was calculated for uncontrolled, PID-controlled, and DRL-controlled water levels during each rainfall event to further evaluate their performance. A comparison of the RMSE values in Table 6 revealed that, although the DRL control had a narrower action space than the PID control with a continuous action space, the DRL control was as effective as the PID control in keeping the water level near the target level during various rainfall events. The RMSE values of the DRL control for four rainfall events were less than or equal to the value for the PID control. The effective control of the groundwater indicated that the agent successfully learned the control policy for managing the water table under different weather conditions.

**Figure 7:**
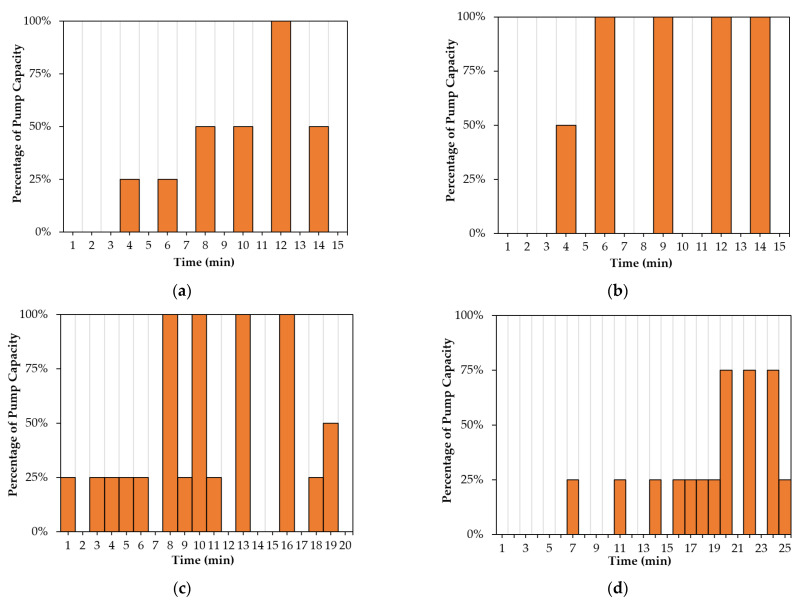
DRL actions during different rainfall events: (**a**) 15 min-constant; (**b**) 15 min-normal; (**c**) 20 min-descending; (**d**) 25 min-ascending.

Figure 7 demonstrates the selection of actions (i.e., 0%, 25%, 50%, 75%, and 100% of the pumping capacity) by the DRL agent for regulating the pump’s flow rate. In all four rainfall events with varying patterns, durations, and total rainfall depths, the DRL agent used a distinct combination of actions to keep the water table close to the target level. The results in this figure indicate that the agent has no bias for taking a specific sequence of actions. Additionally, it was observed that the agent took actions with higher pumping rates (75% and 100%) when the water level was higher and took actions with lower pumping rates (0%, 25%) when the water level was lower.

**Figure 8:**
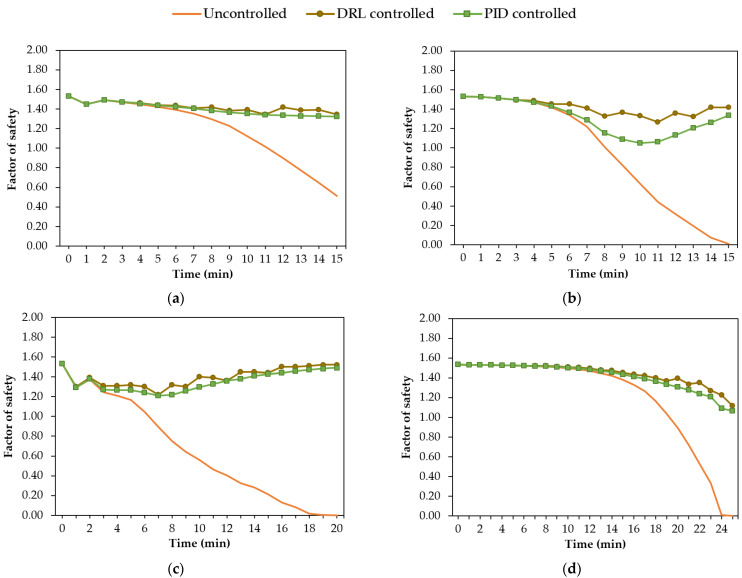
Variation in the slope’s factor of safety obtained for different control methods during rainfall events: (**a**) 15-min-constant; (**b**) 15-min-normal; (**c**) 20-min-descending; (**d**) 25-min-ascending.

Figure 8 shows the results of the slope stability analysis for the DRL-controlled, PID-controlled, and uncontrolled geosystems during four different rainfall events. For the uncontrolled geosystem with no pumping, the FS of the slope dropped from the initial value of 1.53 to a value lower than 1.0, indicating a slope failure during all rainfall events. By contrast, for the DRL-controlled and PID-controlled geosystem, the FS of the slope remained above 1 throughout all rainfall events. It was also observed that the slope’s FS using DRL control experienced smaller variations than using the PID control. The maximum decrease in the FS using the DRL control was 14.3% less than that using the PID control during a 15-min-normal rainfall event, as shown in Figure 8b. Although the reward function for DRL was constructed based on the water head value at a single point, the DRL agent successfully regulated the groundwater and reduced the risk of failure in the slope.

In this study, PID control was used as a benchmark for the proof of concept of the DRL control. Although PID is one of the most widely used control methods, it may suffer from a lack of intelligence and resilience due to its passive nature. The complicated field conditions of real-world geosystems, such as the stochastic nature of precipitation events, may require control with high intelligence and resilience, as found in DRL. In extreme precipitation events, depending on the defined reward function, DRL may start pumping at earlier stages to reduce the groundwater table and help to prevent landslide hazards in the geosystem. PID is a reliable control method for systems that are easy to design, with a known control variable and error value. However, DRL performs based on the good/bad behavior defined as a reward function. For unknown environments, the implementation of DRL may be much easier than that of PID.

## 5. Discussion

In this section, the influence of some key factors in the use of DRL for generating intelligent groundwater control systems is investigated. These factors include (1) the state space size (or the number of observations from the environment), (2) transfer learning (i.e., transfer of knowledge from a pre-trained model to another training with a different rainfall event), and (3) action space size. Additionally, the limitations of the current study and directions for future work are discussed.

### 5.1. Influence of State Space Size

The DRL agent takes action after assessing the state of the environment. Thus, the state space size (i.e., the number of observations) may affect the performance of the agent. Due to this concern, we explored the influence of the number of observations on groundwater control by the DRL agent. Candidate observations from the geosystem (i.e., indicators of system status for DRL) include the water head at any point and the rain intensity at any time. The water head at point “P” must be included in the state space since the reward function was constructed based on the water head at this point. Other observations from the environment could be the rain intensity at the current time and future time steps. The influence of the state space size was investigated using two typical scenarios. One scenario was the state space with only one observation, termed S1 for simplicity, which included the water level at point “P”. The second scenario was the state space with three observations, S3, which included the water level at point “P”, rain intensity at the current time step, and rain intensity at the next time step. The rest of the parameters were identical in both scenarios.

**Figure 9:**
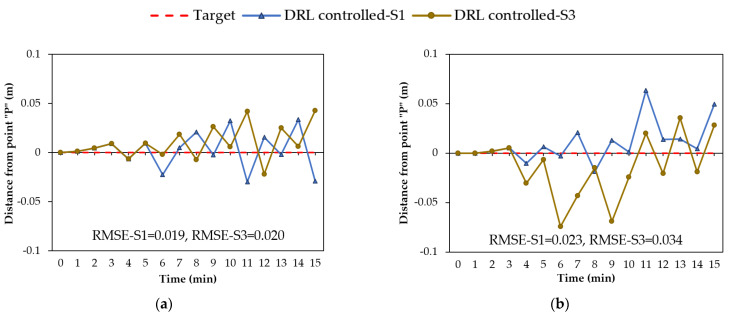

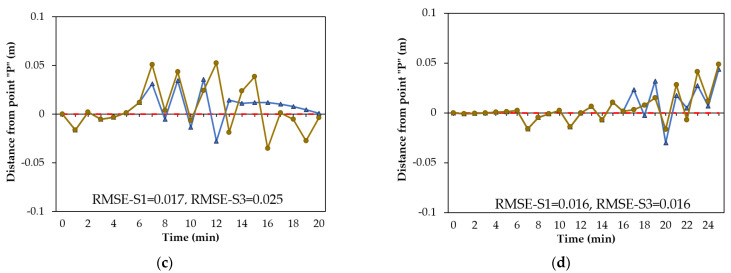
DRL-controlled groundwater with one and three observations (S1 and S3) from the environment during rainfall events: (**a**) 15-min-constant; (**b**) 15-min-normal; (**c**) 20-min-descending; (**d**) 25-min-ascending.

Figure 9 shows the groundwater control in both scenarios (i.e., S1 and S3) during the four rainfall events. In both scenarios, the results of the water head at point “P” showed no failure in controlling the groundwater during various rainfall events. However, a comparison of the RMSE values for S1 and S3 indicated that the performance of the DRL agent in S1 was slightly better, especially in the 15-min-normal and the 20-min-descending rainfall events, as shown in Figure 9b,c, respectively. In these events, the S1 scenario with a state space size of 1 resulted in RMSE values (i.e., 0.023 and 0.017) lower than those of the S3 scenario with a state space size of 3 (i.e., 0.034 and 0.025). The reason is that the DRL agent in S3 took more conservative actions compared to S1. This implies that providing additional information about the geosystem and its future status for the DRL agent, such as the rain intensity at the current time and the next time step in the S3 scenario, can assist in detecting an impending hazard and responding to it sooner by taking conservative actions and lowering the water table. The advantages of the S3 scenario with three observations may not be reflected in the current study, since the goal of this geosystem was to keep the water level near the target level while avoiding unnecessary pumping to conserve energy. If the reward function was constructed in such a way as to incentivize the agent to take more conservative actions for an upcoming intense rainfall event, the agent may lower the groundwater at earlier stages and better manage the flood hazard.

### 5.2. Effectiveness of Transfer Learning

One advantage of adopting RL for geosystem management is that the learning agent can improve the control policy over time during training (through 10,000 episodes here) via the received rewards and penalties. Furthermore, transferring the DRL agent’s gained knowledge from a pre-trained model to another training with a different rainfall event may further improve groundwater control. In this subsection, the impact of transfer learning on groundwater control is investigated.

For this purpose, the DRL agent was initially trained with the 15-min-constant rainfall event. The DQN agent was then trained with the 15-min-normal rainfall event. The weights of the model were initiated using the weights of the pre-trained model with the 15-min-constant rainfall event. Subsequently, the DQN agent was trained with the 20-min-descending rainfall event and the 25-min-ascending rainfall event, respectively. It is noted that the model for each training step was initiated with the weights of the previously trained model. In addition, the state space for all models contained three observations from the environment.

**Figure 10:**
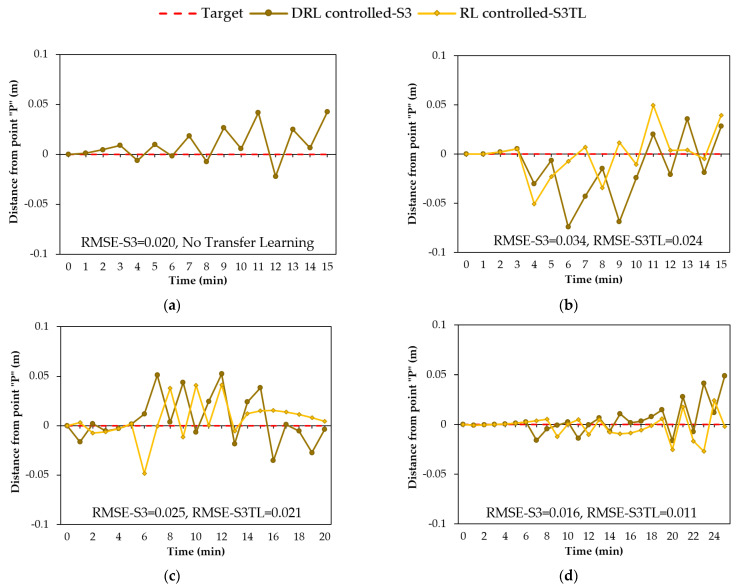
DRL-controlled groundwater with and without the transferred knowledge from a pre-trained model (S3 and S3TL) during rainfall events: (**a**) 15-min-constant; (**b**) 15-min-normal; (**c**) 20-min-descending; (**d**) 25-min-ascending.

To demonstrate the influence of transfer learning, the DRL agent was trained in two ways: without initializing the weights from a pre-trained model (termed S3 for simplicity) and with weights initialized using the previously trained model (termed S3TL). Figure 10 shows groundwater control outcomes for S3 and S3TL during the four types of rainfall events. Figure 10a only includes the result of S3, because the first training step with the 15-min-constant rainfall event had no previously trained model. As shown in Figure 10b–d for the three rainfall events, the RMSE values for training using transfer learning (S3TL) were lower than the values for training without any knowledge (S3). Lower values of RMSE indicated that the training with transfer learning can better regulate the groundwater and keep the water level closer to the target level. Furthermore, by comparing Figure 10a–d, it was observed that the distance from the target level was reduced as the agent gained more experience in controlling the water table during different rainfall events. This investigation confirmed the efficiency of the transferred knowledge in improving the groundwater control as the agent trains with more rainfall events.

### 5.3. Influence of Action Space Size

The number of actions that can be taken by the agent during various rainfall events may impact groundwater control in the geosystem. As a result, the effect of the action space size on groundwater control is investigated in this subsection. For this purpose, two types of control, binary control with a state space size of two (on and off) and intermittent control with a state space size of five (0%, 25%, 50%, 75%, 100%), are studied.

**Figure 11:**
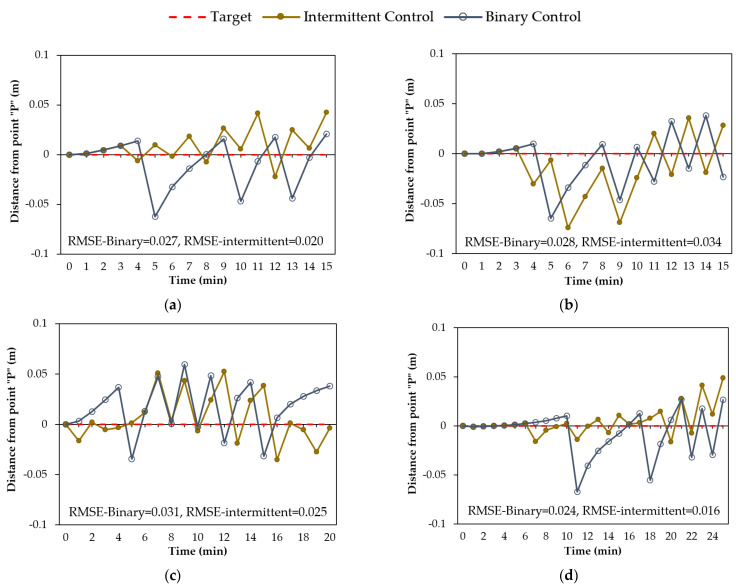
DRL-controlled groundwater using action space sizes of two (binary) and five (intermittent) during rainfall events: (**a**) 15-min-constant; (**b**) 15-min-normal; (**c**) 20-min-descending; (**d**) 25-min-ascending.

**Figure 12:**
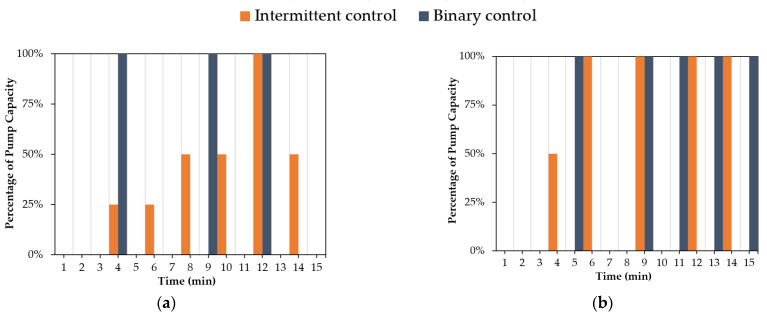

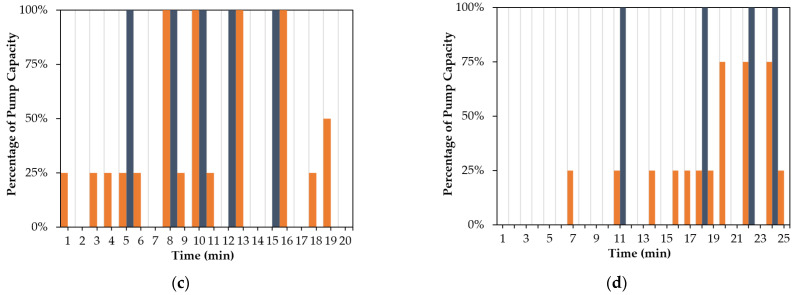
Selection of actions to regulate the pump’s flow rate using binary and intermittent control during rainfall events: (**a**) 15-min-constant; (**b**) 15-min-normal; (**c**) 20-min-descending; (**d**) 25-min-ascending.

Figure 11 displays the results of the water head at point “P” using the binary control and intermittent control in the geosystem during various rainfall events. As shown in Figure 11a,c,d, the RMSE values using the intermittent control are lower than the values using the binary control, except for the 15-min-normal rainfall event shown in Figure 11b. The reason is that this 15-min-normal rainfall event (see Figure 2b) has higher rain intensities compared to the other events (see Figure 2a,c,d). In this case, the DRL agent must regulate the pump with full capacity to control the water table. Figure 12 demonstrates the actions taken by the agent using the binary and intermittent control during different rainfall events. As shown in Figure 12b, the choice of actions for the intermittent control during the 15-min-normal rainfall event demonstrated that the DRL agent mostly selected two actions (0% and 100%), similar to the binary control. By contrast, it can be observed from Figure 12a,c,d that, for the intermittent control, the agent employed actions associated with lower flow rates during lower rain intensities. This selection of actions led to a water level close to the target level, as shown in Figure 11a,c,d. In comparison to binary control, intermittent DRL control of groundwater can enable a more efficient pumping system by operating at lower flow rates when the distance from the target level is small, and thus can better reduce the pumping energy cost in long-term operations.

### 5.4. Limitations and Future Work

Here, the limitations and applications of the current study for future work are discussed. One of the main limitations of implementing DRL is its considerable computational demand for training the agent. Depending on the length of the rainfall events, training for 10,000 episodes in this study took approximately 100–130 h of real-world time. In addition, defining a complex and precise reward function may not be always easy for more complicated tasks. Looking at the future directions of this study, the agent will be trained with more complex field conditions and rainfall patterns. Accordingly, the knowledge gained in this study will be transferred to a physical lab-scale geosystem that will serve as a real-world environment for the DRL agent.

## 6. Conclusions

This study aimed to take a small but significant step toward developing an autonomous geosystem and minimizing the operational costs of groundwater control. This paper studied an intelligent geosystem enabled by deep reinforcement learning (DRL) for controlling the groundwater in slopes subjected to precipitation. The main contributions of this study are (1) modifying the developed DRL framework for the intelligent control of groundwater in a typical geosystem (i.e., a slope equipped with a pump and subjected to rainfall events), (2) evaluating the DRL control of the water level against the traditional proportional-integral-derivative (PID)-controlled and uncontrolled water levels, (3) assessing the performance of DRL control in preventing slope failures, (4) investigating the effectiveness of transferring the DRL agent’s knowledge from a pre-trained model to a new training task with a different rainfall event, (5) exploring the influence of the number of observations from the environment, and (6) investigating the impact of binary control versus intermittent control on the groundwater management. The results showed that the DRL agent learned how to control a pump to lower the water table and mitigate the landslide hazard in the slope. Despite the diverse rainfall patterns, durations, and total rainfall depths, the DRL agent could successfully learn the most effective control policy to keep the water level near the target level and prevent slope failures. Furthermore, the DRL agent improves the groundwater control policy as it is trained with more rainfall events. The findings of this study point out a feasible avenue for developing intelligent geosystems.

## Figures and Tables

**Table 1:** Input parameters for the seepage model.

Definition	Soil
$\mathrm{Saturated}\;\mathrm{hydraulic}\;\mathrm{conductivity},\;K_{S}$ [m/s]	6 × 10^–4^
$\mathrm{Saturated}\;\mathrm{specific}\;\mathrm{storage},\;S_{S}$ [1/m]	1 × 10^–4^
Porosity, n [–]	0.32
$\mathrm{Empirical}\;\mathrm{parameter},\;P_{0}$ [Pa]	1200
Empirical parameter, a [–]	0.6

**Table 2:** Parameters related to pump’s outflux.

Parameter	$Q_{p}$ (m^3^/s)	r (m)	$\chi_{A_{t}=0}$	$\chi_{A_{t}=1}$	$\chi_{A_{t}=2}$	$\chi_{A_{t}=3}$	$\chi_{A_{t}=4}$
Value	0.0002	0.02	0	0.25	0.5	0.75	1

**Table 3:** Tuned PID parameters.

Parameter	$k_{p}$	$k_{i}$	$k_{d}$
Value	0.0088	0.0001	0.0251

**Table 4:** Model parameters for slope stability analysis.

Definition	Soil
$\mathrm{Dry}\;\mathrm{unit}\;\mathrm{weight}\;\mathrm{of}\;\mathrm{soil},\;\gamma_{dry}$ [kN/m^3^]	16.40
$\mathrm{Saturated}\;\mathrm{unit}\;\mathrm{weight}\;\mathrm{of}\;\mathrm{soil},\;\gamma_{sat}$ [kN/m^3^]	19.54
Friction angle, $\phi^{\prime }$ [°]	34°
Cohesion, $c^{\prime }$ [kN/m^2^]	0
Pore air pressure, $U_{a}$ [kN/m^2^]	0
$\mathrm{Number}\;\mathrm{of}\;\mathrm{vertical}\;\mathrm{slices},\;N_{x}$ [–]	35
$\mathrm{Number}\;\mathrm{of}\;\mathrm{cells}\;\mathrm{within}\;\mathrm{the}\;\mathrm{vertical}\;\mathrm{slices},\;N_{c}$ [–]	10

**Table 5:** Hyperparameters for DQN.

Parameter	Value
Number of hidden layers	2
Number of neurons in each hidden layer	25, 25
Number of episodes for training	10,000
Batch size	60
Learning rate, α	10^−3^
Gamma, γ	0.9
Initial epsilon	1
Final epsilon	0.01
Epsilon decay	0.995
Target network update frequency, N	Every 60 iterations
Replay memory size	5000

**Table 6:** RMSE values for the uncontrolled, PID-controlled, and DRL-controlled water levels during various rainfall events.

Control Method	RMSE			
	15 min-constant	15 min-normal	20 min-descending	25 min-ascending
Uncontrolled	0.093	0.145	0.197	0.115
PID-controlled	0.022	0.034	0.028	0.022
DRL-controlled	0.020	0.034	0.025	0.016

## Data Availability

Data will be available upon request.
